# A systematic review of economic evaluations of advanced therapy medicinal products

**DOI:** 10.1111/bcp.14275

**Published:** 2020-03-31

**Authors:** Huw Lloyd‐Williams, Dyfrig A. Hughes

**Affiliations:** ^1^ Centre for Health Economics and Medicines Evaluation Bangor University Wales UK

**Keywords:** cell therapy, cost‐effectiveness, gene therapy, health technology assessment, regenerative medicine

## Abstract

**Aims:**

Advanced therapy medicinal products (ATMPs) represent a new category of medicinal products with a potential for transformative improvements in health outcomes but at exceptionally high prices. Routine adoption of ATMPs requires robust evidence of their cost‐effectiveness.

**Methods:**

A systematic literature review of economic evaluations of ATMPs, including gene therapies, somatic cell therapies and tissue‐engineered products, was conducted. Literature was searched using MedLine, Embase, PubMed, Cochrane Register, the NHS Economic Evaluation Database and the grey literature of health technology assessment organisations with search terms relating to ATMPs and economic evaluations. Titles were screened independently by 2 reviewers. Articles deemed to meet the inclusion criteria were screened independently on abstract, and full texts reviewed. Study findings were appraised critically.

**Results:**

4514 articles were identified, of which 23 met the inclusion criteria. There was some evidence supporting the cost‐effectiveness of: chimeric antigen receptor T‐cell therapy axicabtagene–ciloleucel (Yescarta), embryonic neural stem cells, tumour infiltrating lymphocytes, in vitro expanded myoblast, autologous chondrocyte implantation, ex vivo gene therapy (Strimvelis) and voretigene neparvovec (Luxturna). However, estimates of cost‐effectiveness were associated with significant uncertainty and high likelihood of bias, resulting from largely unknown long‐term outcomes, a paucity of evidence on health state utilities and extensive modelling assumptions.

**Conclusion:**

There are critical limitations to the economic evidence for ATMPs, most notably in relation to evidence on the durability of treatment effect, and the reliability of opinion‐based assumptions necessary when evidence is absent.

## 1 INTRODUCTION

Advanced therapy medicinal products (ATMPs), which include gene therapies, somatic cell therapies and tissue‐engineered products have the potential for transformative improvements in health outcomes for a wide range of diseases, including certain cancers, neurodegenerative and cardiovascular diseases.^1^, ^2^ Clinical application of somatic cell therapies and tissue‐engineered products is frequently referred to as *regenerative medicine.* The number of ATMPs being approved is rising^3^ and, given their high cost, there is a pressing need for robust economic evidence of these therapies in order to inform decisions made by healthcare payers.

ATMPs pose specific challenges in evidence generation, health technology assessment (HTA) and financing.^4^ A key feature of ATMPs is their price, which can in some instances exceed £1m per patient. Such high (often up‐front) costs make ATMPs particularly problematic in terms of meeting usual thresholds of cost‐effectiveness and being affordable to healthcare payers. Moreover, there may be methodological challenges, such as in relation to uncertainty in the evidence of the effectiveness of newly approved ATMPs; the nature of the distribution of costs in relation to the accrual of benefits, and how these are affected by choice of discount rates; whether curative treatments may be considered differently to treatments that create smaller incremental benefits; and consideration of value attributes that may not be captured adequately in the quality‐adjusted life year (QALY).

The National Institute for Health and Care Excellence (NICE) in the UK suggested that a completely new reference case is not needed. Their mock economic evaluation of a chimeric antigen receptor (CAR) T‐cell therapy accepted existing methods of economic evaluation as being *fit for purpose* in the evaluation of ATMPs.^5^ More recently, the independent US‐based Institute for Clinical and Economic Review following a review in collaboration with NICE and the Canadian Agency for Drugs and Technologies in Health, published adaptations to its value assessment framework for potential cures and other treatments that qualify as high‐impact *single or short‐term therapies.* ^6^ Marsden et al. (2019)^7^ suggested new analytic approaches are required, suggesting that “patients with rare genetic diseases, along with the gene replacement therapies they use, present a unique set of conditions that warrant equally unique analytic approaches to estimating value for money.” Similarly, Drummond et al. (2019)^8^ suggested that some unique characteristics need to be taken into account.

The aim of this study was to review and critique published economic evaluations of ATMPs, in order to: (i) highlight current evidence on the cost‐effectiveness of ATMPs; (ii) identify specific methodological challenges; and (iii) assess how these challenges were approached by analysts.

## 2 METHODS

### 2.1 Protocol, registration and reporting

The protocol for this review was registered with the International Prospective Register of Systematic Reviews (PROSPERO, reference CRD42019125069). The review is reported in accordance with the Preferred Reporting Items for Systematic Reviews and Meta‐Analyses (PRISMA) statement.^9^

### 2.2 Review question

The principal review question was: what are the main challenges and solutions for the economic evaluation of ATMPs?

### 2.3 Search strategy

We searched the literature using MedLine, Embase, PubMed, Cochrane Central Register of Controlled Trials, National Health Service Economic Evaluation Database, Health Technology Assessment Centre for Reviews and Dissemination, and Web of Science, for relevant articles published from database inception up to April 2019. The search strategy involved combining terms for ATMPs and economic evaluations using the Boolean ‘AND’ operator. The search was restricted to studies of human subjects and written in the English language. An additional search of the *grey* literature contained within the websites of HTA organisations was conducted. Further articles were identified from other related systematic reviews and reference lists of included studies. The full search strategy is detailed below.

(Strimvelis [tw] OR “Autologous chondrocyte implantation” [tw] OR Imlygic [tw] OR Luxturna [tw] OR Yescarta [tw] OR Kymriah [tw] OR tisagenlecleucel [tw] OR “chimeric antigen receptor” [tw] OR CAR‐T [tw]) OR Gencidine [tw] OR Oncorine [tw] OR Neovasculgen [tw] OR Zalmoxis [tw] OR tonogenchoncel‐L [tw] OR GS010 [tw] OR NSR‐REP1 [tw] OR “valoctocogene roxaparvovec” [tw] OR AMT‐061 [tw] OR AVXS‐101 [tw] OR Generx [tw] OR RT‐100 [tw] OR Pexa‐Vec [tw] OR Collategene [tw] OR VM202 [tw] OR “LentiGlobin BB305” [tw] OR Lenti‐D [tw] OR GSK2696274 [tw]) AND (economics [mh] OR “health technology assessment” [tw]) AND english [la].

### 2.4 Eligibility criteria/study selection

Economic evaluations of ATMPs, reported in full, published in the past 20 years (2000–2019) and in the English language were included. Only full economic evaluations were included (i.e. cost effectiveness, cost utility or cost benefit analyses). Partial economic evaluations (e.g. cost minimisation or cost consequence analyses) were excluded, as were studies only reporting the burden of disease or cost of illness. We excluded editorials, letters, historical articles, discussion or commentary articles, and evaluations published only as abstracts.

### 2.5 Data extraction

Identified articles were screened by 2 reviewers independently according to the exclusion and inclusion criteria; first by title, followed by abstract, and finally by full article text. Any discrepancies were resolved in discussion with the third reviewer. Extracted data included year and country of publication, clinical indication, ATMP and comparator, method of economic evaluation, time horizon, total intervention and comparator costs, QALY gain, incremental cost‐effectiveness ratios (ICERs), results of sensitivity analyses, principal study findings, issues of generalisability, study limitations and key methodological challenges as reported by the authors of each study.

### 2.6 Quality of reporting assessment

Articles were assessed for their quality of reporting by their compliance with the Consolidated Health Economic Evaluation Reporting Standards.^10^ Studies were scored against each of the 24 checklist items according to whether reporting *fully satisfied* or *did not satisfy* the item requirements. The overall quality of reporting was presented as a percentage score of applicable items. Studies scoring above an arbitrary threshold of 75% were considered to be of higher reporting quality. The quality of reporting of individual items from the checklist is expanded further in the narrative.

### 2.7 Narrative synthesis

A narrative synthesis of the methodological challenges associated with economic evaluations of ATMPs was carried out following the methods of Nagpal et al. (2019),^11^ and based on the information extracted and judgements made on study quality. This approach synthesises findings from multiple studies and uses the words and text from these studies to produce a summary and explanation of the findings therein.

## 3 RESULTS

### 3.1 Search results

**FIGURE 1:**
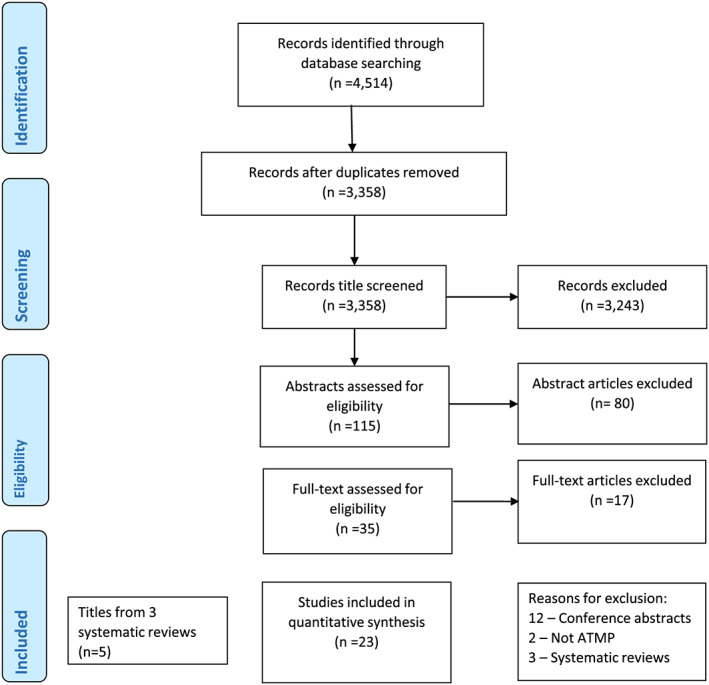
PRISMA flowchart for this review. ATMP, advanced therapy medicinal product

In total, 4514 studies were identified following the initial search. Removal of duplicates resulted in 3358 potentially relevant articles. Title screening resulted in 115 papers, which further reduced to 35 following abstract screening, and 18 following the review of full article texts. The reasons for exclusion are given in Figure 1. Five additional papers were identified from other sources, resulting in 23 studies being included in the review. The data extracted from the included studies are presented in Tables 1, 2, 3.

**TABLE 1:** Principal characteristics of included studies

Reference	Year	Country (currency)	Clinical indication	ATMP	Comparator	Method	Time horizon
**Somatic‐cell therapy medicines**							
^5^	2017	UK (GBP £)	Acute lymphoblastic leukaemia	CAR T‐cell therapy (unspecified)	Standard of care	CUA (conventional assessment of cost‐effectiveness at the patient level)	Lifetime
^12^	2006	Sweden (euro €)	Parkinson's disease	Embryonic neural stem cells	Standard pharmacological therapy	CUA (Markov state transition model)	25 years
^13^	2017	Netherlands (euro €)	Metastatic melanoma	Tumour infiltrating lymphocytes	Ipilimumab	CUA (Markov decision model)	Lifetime
^14^	2019	USA (USD $)	Relapsed/refractory B‐cell acute lymphoblastic leukaemia	CAR T‐cell therapy (unspecified)	Standard of care	CUA (microsimulation model)	n/a
^15^	2018	Scotland (GBP £)	Relapsed or refractory diffuse large B cell lymphoma and primary mediastinal large B cell lymphoma	Axicabtagene–ciloleucel	Best supportive care	CUA (3‐state partitioned survival model)	Lifetime
^16^	2019	Scotland (GBP £)	Relapsed or refractory diffuse large B‐cell lymphoma	Tisagenlecuecel	Salvage chemotherapy regimens	CUA (cohort‐based partitioned survival model)	46 years
^17^	2012	Sweden (euro €)	Stroke	Intracerebral stem cell implantation	Standard post stroke care	CUA/CBA (decision tree model)	Lifetime
^18^	2010	UK (GBP £)	Multiple sclerosis	Autologous haematopoietic stem cell transplantation	Mitoxantrone	CUA (Markov modelling)	1 year
^19^	2018	USA (USD $)	Childhood B‐cell acute lymphoblastic leukaemia	Tisagenlecleucel	Clofarabine	CUA (decision tree and long‐term semi‐Markov partitioned survival model)	Lifetime
^20^	2018	Denmark (euro €)	Female stress urinary incontinence	In vitro expanded myoblast	Midurethral slings	CEA (decision tree)	5 years
^21^	2018	USA (USD $)	Paediatric patients with relapsed or refractory leukaemia	Tisagenlecleucel	Clofarabine	CEA (decision analytic model)	Lifetime
^22^	2018	USA (USD $)	Relapsed or refractory paediatric B‐cell acute lymphoblastic leukaemia	Tisagenlecleucel	Blinatumomab, clofarabine combination therapy	CUA (Markov modelling)	Lifetime
^23^	2018	USA (USD $)	Relapsed or refractory large B‐cell lymphoma	Axicabtagene–ciloleucel	Salvage chemotherapy	CUA (decision model)	Lifetime
**Tissue engineered medicines**							
^24^	2018	Norway (euro €)	Focal cartilage defects in the knee	Autologous chondrocyte implantation	Microfracture	CEA (decision tree)	5 years
^25^	2010	Belgium (euro €)	Knee cartilage lesions	ChondroCelect used in ACI	Microfracture	CUA (decision tree)	40 years
^26^	2007	UK (GBP £)	Urethral defects and bladder resection for cancer	Tissue engineering	Ileocystoplasty	CEA (headroom method)	n/a
^27^	2017	UK (GBP £)	Chondral defect in the knee	Autologous chondrocyte implantation	Microfracture	CUA (Markov state transition model)	Lifetime
^28^	2012	USA (USD $)	Articular cartilage injury	ACI collagen patch	ACI periosteal patch	CUA (decision analytic model)	Lifetime
^29^	2018	Netherlands (euro €)	Articular cartilage repair	Instant MSC product accompanying autologous Chondron transplantation (IMPACT)	MicroFracture & Autologous chondrocyte implantation	CEA (decision tree)	Lifetime
**Gene therapy medicines**							
^30^	2018	USA (USD $)	Haemophilia	Gene therapy	Prophylaxis with factor VIII	CUA (Markov state transition model)	10 year
^31^	2017	UK (GBP £)	Adenosine deaminase deficiency–severe combined immunodeficiency	Strimvelis haematopoietic stem cell transplant	Matched unrelated donor	CUA (decision tree)	n/a
^32^	2018	UK (GBP £)	Adenosine deaminase deficiency–severe combined immunodeficiency	Strimvelis	Haematopoietic stem cell transplant	CUA (decision tree & Markov modelling)	Lifetime
^33^	2019	USA (USD $)	Biallelic RPE65‐mediated inherited retinal disease	Voretigene neparvovec	Standard of care	CUA (2 state Markov model)	20 years

ATMP, advanced therapy medicinal product; CAR, chimeric antigen receptor; ACI, autologous chondrocyte implantation; MSC, allogeneic mesenchymal stromal (stem) cells; CUA, cost‐utility analysis; CBA, cost–benefit analysis; CEA, cost‐effectiveness analysis

**TABLE 2:** Main results of included studies

Reference	Total intervention cost	Total comparator costs	QALY gain	ICER^1^/cost per point improvement in outcome^*^/Headroom^2^	Sensitivity analysis
**Somatic‐cell therapy medicines**					
^5^	£449 128	£75 962	8.82–1.36 = 7.46	£49 995^1^	If the discount rate for costs and outcomes was reduced to 1.5% then the cost per QALY would be reduced to £35 162.
^12^	HY stage III: €156 467 HY stage IV: €163 588	HY stage III: €158 943 HY stage IV: €186 279	HY stage III: 0.873 HY stage IV: 1.133	Intervention cost saving	Univariate analysis: Time horizon (10, 20, 30 years); discount rate (0%, 5%); treatment efficacy (±50%); occurrence of complications (±100%); analytical perspective (direct medical costs only *vs* including other direct costs); method of determining utilities. The ICER was cost saving for most variables with the exception of postoperative disease progression, where it was cost increasing
^13^	€62 000	€91 487	0.07	Intervention dominates ICER n/a	The parameters with the most impact on the incremental costs were survival, drop‐outs and costs of treatment. For the incremental QALYs, these were survival and utilities.
^14^	$968 800	$440 600	16.76–8.58 = 8.18	$64 600^1^	If the 1‐year survival dropped below 57.8% then the ICER rose above $100 000 per QALY, and CAR T‐cell therapy would not be considered cost effective.
^15^	£1 035 601	£405 126	31.3–22.8 = 8.5	£74 430^1^	No sensitivity analysis performed.
^16^	Not reported	Not reported	4.1	£57 943	The results are associated with increased uncertainty when key variables in the model were revised.
^17^	$202 901	$221 956	1.34	Intervention is cost saving	Univariate analysis: relative efficacy of SCT; mode of transplantation; age at stroke onset; annual risk of recurrent stroke; SCT procedure risk of death; intervention on mRS3/4; extended leave period. The highest ICER came with intervention on mRS 4.
^18^	£131 666	£107 126	4.1–5.12 = −1.02	Intervention is dominated	Univariate analysis: Transplant related mortality rate (0/1.3%); relative PFS hazard ratio between HSCT and mitoxantrone; tariff cost of HSCT (±25%), costs of managing multiple sclerosis (±25%); discount rate (0/3.5%). The ICER is most sensitive to the cost of transplantation itself.
^19^	$666 754	$337 256	9.28–2.10 = 7.18	$45 871^1^	Uncertainty around long‐term survival was explored through variation in the discount rate used in the sensitivity analysis
^20^	€2224	€1223	0.11	Negative ICER. Intervention dominated by comparator	One‐way sensitivity analysis based on the upper limit cure rate for in vitro expanded myoblasts indicates that this may become more effective as compared with the standard midurethral slings procedure.
^21^	$667 000	$337 000	9.28	$46 000^1^	Across scenario analyses that included more conservative assumptions regarding long‐term relapse and survival, the ICER ranged from $37 000 to $78 000 per QALY gained.
^22^	$599 000	$374 000	12.1	$61 000^1^	In probabilistic sensitivity analyses, tisagenlecleucel at a 5‐year relapse‐free survival rate of 40% was cost effective in 99.3, 98.7 and 6.0% of simulations at willingness to pay thresholds of $150 000, $100 000 and $50 000, respectively
^23^	$552 921	$172 737	7.67–1.13 = 6.54	$55 128^1^	Scenario analyses in which patients in remission had mortality rates 10 % and 20% higher than the age‐matched general US population. Cost‐effectiveness was most sensitive to the fraction achieving long‐term remission, discount rate and axicabtagene–ciloleucel price.
**Tissue engineered medicines**					
^24^	€14 238	€4329	Not reported	€2134^*^	A 66% reduction in the total costs following ACI or a 190% increase in the total costs of microfracture led to equivalent total costs at 5 years
^25^	€24 879	€1035	1.282	€16 229^1^	Probabilistic sensitivity analysis showed that 80% of simulations were below a threshold of €22 000 per QALY
^26^	Not reported	Not reported	Not reported	£16 268^2^	n/a
^27^	£17 740	£3020	n/a	£14 395^1^	Cost of cells for ChondroCelect were £16 000. Sensitivity analysis was conducted to vary this figure by reducing the costs by 25, 50 and 75%. The time horizon was also varied by 10, 20, 30, 40 and 50 years. The cost of cells are a key driver for the ICER.
^28^	$66 752	$66 939	0.07	$9466 (average cost‐effectiveness ratio)	Sensitivity analysis was performed regarding the additional cost of the type I/III collagen patch ($780) in ACI‐C as well as the rate of graft hypertrophy after ACI‐P (25%). Small changes in outcome affects the ICER substantially so that ACI‐P becomes more cost effective if the utility value of patients doing well after ACI‐P is increased slightly from 0.85 to 0.86 or that of ACI‐C is decreased slightly from 0.85 to 0.84.
^29^	€11 797	€6081 (MF)	0.04	€610 600^1^	If the utilities of IMPACT were 10% lower than ACI, the maximum costs of IMPACT would be €23 697
**Gene therapy**					
^30^	$1 022 049	$1 693 630	8.33–6.62 = 1.71	Intervention dominates ICER n/a	Only variation of gene therapy cost caused the gene therapy strategy to be no longer cost saving compared with prophylaxis
^31^	Not reported	Not reported	13.6	£36 360^1^	NICE evidence review group proposed a list of changes to be included as a sensitivity analysis. These increased the ICER from the company base case to £86 815 per QALY gained.
^32^	Not reported	Not reported	n/a	£49 975^1^	The results are associated with increased uncertainty when key variables in the model were revised.
^33^	$1 039 000	$213 400	1.3	$480 100^1^	For different levels of visual ability, the ICER and the necessary discount to reach a defined willingness to pay threshold was calculated. The ICER decreased with increasing visual ability at baseline.

QALY, quality‐adjusted life year; ICER, incremental cost‐effectiveness ratio; HY, Hoehn and Yahr (scale); CAR, chimeric antigen receptor; SCT, stem cell transplant; mRS, modified Rankin scale; PFS, progression‐free survival; HSCT, haematopoietic stem cell transplantation; ACI, autologous chondrocyte implantation; ACI‐C, collagen‐covered ACI; ACI‐P, periosteum‐covered ACI; IMPACT, instant allogeneic mesenchymal stromal cells product accompanying autologous chondron transplantation

**TABLE 3:** Principal findings, issues of generalisability, limitations and methodological challenges of included studies as reported by study authors

Reference	Study findings	Generalisability	Limitations	Key methodological difficulties
**Somatic‐cell therapy medicines**				
^5^	Main purpose was to report the potential cost‐effectiveness of CAR T‐cell therapy; and to highlight key uncertainties surrounding these results.	Not reported.	This exercise was conducted on theoretical data and assumed costs, and may not capture the problems associated with real‐world data.	Although evidence about ATMPs is expected to be associated with uncertainty in determining the long‐term costs and benefits to patients and the NHS, existing methods available to estimate the implications of this uncertainty are sufficient. Challenges include: the potential curative nature and claims of long‐term/lifetime benefits; the potentially rapid changes that may arise in product characteristics over time; potential longer‐term patient safety issues because of persistence; organisational and scaling issues; and the potentially significant upfront costs that may arise.
^12^	Long‐term cost savings in most instances in early onset Parkinson's disease patients in HY stages III‐IV.	The model was based on the Swedish health care system, but devised to be applicable to available data on treatment costs and health state utilities for different HY stages. Such data are now available from a variety of countries.	Small number of patient‐level data; clinical effectiveness data based on open‐label transplantation trials	The frequent use of placebo as a comparator, together with the extra attention given to randomised control trial patients may contribute to nonrepresentative outcomes. Use of real‐life observations claimed to be less restricting to allow hypothetical comparisons between standard therapy and a range of different alternatives.
^13^	Tumour infiltrating lymphocytes are expected to generate more QALYs than its comparator at a lower cost and so dominates.	The prices of treatments vary substantially between countries. This reduces the generalisability of the results.	No clinical trial data available and therefore data on the effectiveness of tumour infiltrating lymphocytes had to be drawn from various sources.	It is unknown which patient subgroup had the best response to tumour infiltrating lymphocytes.
^14^	CAR T‐cell therapy increased overall cost by $528 200 and improved effectiveness by 8.18 QALYs, which produced an ICER of $64 600 per QALY per payer perspective. Cost effectiveness was established in 94.8% of iterations at a willingness to pay of $100 000 per QALY.	Not reported.	CAR T‐cell therapy is a new therapy and thus long‐term data on survival, costs, role of HSCT after CAR‐T, and complications that could influence these cost effectiveness analysis results are lacking. Model inputs including costs and utilities from heterogeneous sources.	Used a microsimulation model rather than a Markov model, permitting more complex model design than traditional Markov models.
^15^	As axicabtagene–ciloleucel is an ultraorphan medicine, Scottish Medicines Consortium can accept greater uncertainty in the economic case, despite a base case ICER of £57 943 per QALY gained.	Not reported.	The absence of any directly comparative data.	Longer‐term data are required to confirm whether axicabtagene–ciloleucel is a curative treatment.
^16^	The intervention produced an ICER of £49 975 per QALY gained when compared to chemotherapy regimen gen‐ox, which is under the NICE £50 000 threshold	Not reported.	Haematological malignancy research network data were used to estimate overall survival for chemotherapy patients meaning that a naïve indirect comparison was used as the basis of the estimation of clinical outcomes in the economic model.	An assumption was made that that patients who were alive at 24 months were effectively cured.
^17^	A potential for long‐term cost savings by reducing the disability after stroke; societal value up to US $166 500 (US $184 567), particularly in younger patients with stroke with moderate disability, with possible cost effectiveness estimated down to relative efficacy of 14%.	Enables cost–benefit analysis for patients with stroke under a wide range of assumptions	Effectiveness of SCT was based on expert opinion; did not include differential costs of early *vs* late administration poststroke; limited standard care data reflecting survival, treatment patterns, and transition probabilities for mRS.	Ideally health economic analyses are based on long‐term data. If not available, and for most treatments only short‐term data are available, disease modelling provides a way of estimating long term effects.
^18^	A potential to achieve a level of cost effectiveness that is acceptable to policymakers and health care purchasers, but is largely determined by the interpretation of available clinical effectiveness data and the duration over which such effects may be observed.	The focus of the analysis was on the potential cost effectiveness of autologous HSCT in the management of secondary progressive multiple sclerosis only.	The absence of direct randomised controlled trial evidence to input into the model.	Modelling cannot be considered a substitute for good quality clinical trial evidence.
^19^	Total cost for tisagenlecleucel was double that of clofarabine, while the gains in QALYs of tisagenlecleucel was 4× that of clofarabine. The probability of cost‐effectiveness at $50 000 per QALY was about 0.7.	Cost perspective specific to US payer which may not be generalisable to other settings.	This analysis was limited primarily by the lack of comparative evidence available for these therapies. Evidence on long‐term effectiveness is still unknown, which resulted in assumptions being made related to trial survival curve extrapolation and the time point at which long‐term survivors would be considered effectively cured.	The authors closely followed the methodology used in the ‘curative intent’ mock evaluation of CAR T‐cell therapy.^5^ The differences in estimates between the 2 models are probably due to the use of 2 different approaches to curve extrapolation.
^20^	IVM is dominated by MUS treatment but as costs of cell expansion are likely to reduce in the future this may reduce the cost of the IVM procedure.	Using QALYs based on the same multiattribute health status classification system internationally would aid generalisability.	Lack of uniform reporting tools to define outcome of stress urinary incontinence interventions. When robust evidence was not available, the estimates relied on expert opinions.	Concerns about the sensitivity of generic multiattribute health outcomes measures in the context of urinary incontinence.
^21^	The cost‐effectiveness is probably between $37 000 and $78 000 per QALY gained over a patient's lifetime horizon.	Not reported.	Lack of evidence for the comparator, which affects the calculation of the ICER. Due to limited follow up, assumptions had to be made about long‐term survival and when a patient is effectively cured.	Flattening in the tail of the survival curves was observed for both tisagenlecleucel and clofarabine. Standard parametric models probably underestimate survival when flattening in the tail exists; therefore, they used a flexible parametric model to account for this flattening.
^22^	Reduction of the price of tisagenlecleucel to $200 000 or $350 000 would allow it to meet a $100 000 or $150 000 per QALY willingness‐to‐pay threshold in all scenarios.	Not reported	No high‐quality long‐term clinical outcomes data exist for tisagenlecleucel	The authors addressed the main limitation by modelling multiple long‐term effectiveness scenarios, including 1 where all patients eventually experience relapse.
^23^	The likelihood that axicabtagene–ciloleucel is cost‐effective was 95% at a willingness to pay of $100 000 per QALY.	Not reported	The current data of the ZUMA‐1 trial are limited at a median follow up of 15.4 months.	As this analysis used axicabtagene–ciloleucel 1‐year follow‐up data, the authors find it prudent to re‐examine cost effectiveness after additional follow‐up.
**Tissue engineered medicine**				
^24^	For all measures, a 1‐point increase in clinical scores had lower costs for microfracture than for ACI at 5 years.	Unit prices came from a single orthopaedic hospital, which may limit the generalisability of the findings.	Small study population leading to bias. MF group had slightly smaller lesions meaning that they are more responsive to physiotherapy.	Clinical uncertainty limits robustness of economic analysis.
^25^	ChondroCelect shown to be a cost‐effective strategy compared with microfracture and the ICER is below the NICE threshold.	Not reported.	Absence of firm data on the probability and time to occurrence of osteoarthritis TKR. Therefore, a Markov model was not possible.	When the need for TKR increases, ICER expected to decrease in favour of ChondroCelect. Due to higher discount rates for costs rather than effects, the procedure resulting in more TKR patients would also generate more QALYs. However, for the patient the optimal treatment is 1 that minimises pain and discomfort and avoids the need for TKR. Long‐term data are needed to characterise specific events.
^26^	The headroom for tissue‐engineered bladder was estimated at around £16 268. However, the market size is limited reducing potential profitability.	Not reported.	Not reported.	The headroom method is claimed to inform decisions without the need for complex modelling, which may have very wide parameter uncertainty. In the case of a technology yet to be developed, or in early stages of development, the very nature of the product is uncertain, leading to difficulties in its economic evaluation; although the method proposed is a simple cost utility analysis.
^27^	If the decision‐maker is willing to pay £20 000 for a QALY, ACI is 56–59% more likely to be cost‐effective than microfracture.	Not reported.	The length of clinical trial follow‐up was too short and hence, there are no long‐term data on the success and failure rates. Because of the paucity of data from clinical studies, transition probabilities were not available for each transition in the model.	There is a clear lack of evidence on health state utility values for patients that have had cartilage defects of the knee.
^29^	IMPACT can be dominant to ACI over a 5‐year horizon in terms of cost effectiveness	All costs were derived from the hospital administration data and/or from other Dutch data resources, which may limit its transferability to other settings.	Patients included in these models, who reflect randomised controlled trial populations, are not always typical of patients seen in orthopaedic sports practice.	Included only a small number of patients from a randomised controlled trial with a follow‐up of 5 years. Greater patient numbers and a longer follow‐up period will make such an early analysis more reliable.
**Gene therapy**				
^27^	Treatment with gene therapy is likely to be cost saving for the treatment of severe haemophilia A compared with the current standard of care with factor VIII prophylaxis.	Age is an important variable in potentially curative treatments. The results are generalisable to different age groups because altering the probability of death, a good approximation for changes in age, did not significantly alter the cost‐effectiveness of gene therapy.	The assumption that successful gene therapy results in full quality of life could potentially bias results toward gene therapy. The lack of commercially available gene therapy for haemophilia A. limiting the time frame to 10 years reduces the cost‐effectiveness of gene therapy significantly.	The assumption that gene therapy leads to full quality of life could potentially bias the results towards gene therapy.
^13^	The ICER for Strimvelis is below the £100 000 per QALY cost‐effectiveness threshold for highly specialised technologies.	Not reported.	Quality of life data had to be collected from the literature.	Discount rate was 1.5% per annum as the treatment comes under the definition NICE uses for a treatment that restores people to full or near‐full health when they would otherwise die.
^17^	The most plausible ICERs were lower than £100 000 per QALY gained and that Strimvelis should be recommended for treatment of ADA‐SCID where a matched related donor is unavailable.	Not reported.	Given the rarity of the disease, there were some issues with the representativeness of the population that had received Strimvelis to the eligible population in England.	While there is a well‐developed methodological literature for evaluating randomised controlled trials in much larger patient populations, there is less guidance on assessing study designs most appropriate for evaluating specialised technologies in rare conditions.
^33^	The high ICER is driven by the high cost of voretigene neparvovec and the relatively low gains in QALYs. Voretigene neparvovec does not improve survival and is not *curativewk.* QALY gains come from quality of life improvements.	Not reported.	Used utility values from other retinal disease population as quality of life data for RPE65‐mediated retinal disease does not exist. This may have led to biased outcomes.	Without long‐term data, it cannot be known how long benefit will be maintained.

CAR, chimeric antigen receptor; ATMP, advanced therapy medicinal product; HSCT, haematopoietic stem cell transplantation; SCT, stem cell transplant; IVM, in vitro expanded myoblasts; MUS, midurethral slings; ACI, autologous chondrocyte implantation; TKR, total knee replacement; IMPACT, instant allogeneic mesenchymal stromal cells product accompanying autologous chondron transplantation; ADA‐SCID, adenosine deaminase severe combined immunodeficiency; HY, Hoehn and Yahr (scale); mRS, modified Rankin scale; NHS, National Health Service; QALY, quality‐adjusted life year; ICER, incremental cost‐effectiveness ratio

### 3.2 Study characteristics

The review identified economic evaluations of the following ATMPs: CAR T‐cell therapies tisagenlecleucel (Kymriah) and axicabtagene–ciloleucel (Yescarta), embryonic neural stem cells, tumour infiltrating lymphocytes (TIL), in vitro expanded myoblast (IVM), autologous chondrocyte implantation (ACI), autologous CD34+ cells transduced with a lentiviral vector containing the human adenosine deaminase gene (Strimvelis), and voretigene neparvovec (Luxturna).

The main clinical indications included acute lymphoblastic leukaemia, Parkinson's disease, haemophilia, defects of the bladder, knee cartilage lesions, adenosine deaminase deficiency, melanoma, stroke, multiple sclerosis and retinal disease.

Of the identified papers, 16 were cost–utility analyses^5^, ^12^, ^13^, ^14^, ^15^, ^16^, ^17^, ^18^, ^19^, ^22^, ^23^, ^25^, ^27^, ^28^, ^30^, ^31^, ^32^, ^33^ and 5 were cost‐effectiveness analyses.^20^, ^21^, ^24^, ^26^, ^29^ Most studies used some form of economic modelling, mainly Markov models (8 studies),^12^, ^16^, ^17^, ^18^, ^19^, ^25^, ^30^, ^31^ but also decision trees,^17^, ^19^, ^20^, ^21^, ^22^, ^23^, ^24^, ^25^, ^28^, ^29^, ^31^, ^32^ microsimulation,^14^ survival modelling^15^, ^16^ or the headroom method.^26^

The time horizon of included studies varied from 1 year, to lifetime in 12 studies which extrapolated costs and outcomes beyond the available clinical evidence.

### 3.3 Principal study findings

#### 3.3.1 Somatic‐cell therapy medicines

There were 8 economic evaluations of CAR T‐cell therapies, of which 6 suggested they were cost effective. As a bridge to haematopoietic stem cell transplantation, and adopting the recommended methods of NICE, Hettle et al. (2017)^5^ estimated an ICER of £49 995 per QALY gained, which exceeds the usual NICE threshold range for cost‐effectiveness. Sarkar et al. (2019)^14^ found that CAR T‐cell therapy (unspecified) for relapsed/refractory B‐cell acute lymphoblastic leukaemia increased overall cost by US$528 200 and improved outcomes by 8.18 QALYs, resulting in an ICER of $64 600 per QALY gained from a US payer perspective. Cost effectiveness was established in 94.8% of iterations at a willingness to pay $100 000 per QALY. In Tice et al. (2018)^19^ the probability of cost‐effectiveness of tisagenlecleucel for childhood B‐cell acute lymphoblastic leukaemia at US$50 000 per QALY was just over 70%. These were consistent with Whittington et al. (2018),^21^ who estimated an ICER in the range of US$37 000 to $78 000 per QALY gained. The Scottish Medicines Consortium (SMC)^15^ appraised the manufacturer's submission of axicabtagene–ciloleucel, which had an ICER of £57 943 per QALY gained and, given its ultra‐orphan status, accepted the greater uncertainty in the economic case. Roth (2018)^23^ also assessed axicabtagene–ciloleucel and found it to be a potentially cost‐effective alternative to salvage chemotherapy. The SMC's appraisal of tisagenlecleucel (Kymriah)^16^ identified an ICER of £49 975 per QALY gained, and was not considered cost‐effective.

Other economic evaluations of cell‐based therapies include a cost utility analysis by Hjelmgren et al. (2006)^12^ who claimed that embryonic neural stem cells were cost saving in patients with early‐onset Parkinson's disease. Retel et al. (2017)^13^ report that TIL is expected to generate more QALYs than its comparator at a lower cost and so is dominant. Intracerebral stem cell implantation in stroke patients was found to be cost saving by Svensson et al. (2012),^17^ under the assumption that stem cell therapy promotes functional recovery in stroke, improves quality of life and reduces societal costs. Tappenden et al. (2010)^18^ found that autologous haematopoietic stem cell transplantation had the potential to achieve a level of cost effectiveness that is acceptable to policymakers and health care purchasers, but is largely determined by the interpretation of available clinical effectiveness data and the duration over which such effects may be observed. Vilsboll et al. (2018)^20^ found IVM to be dominated by midurethral sling treatment (the comparator) but speculated that the cost of the IVM procedure would reduce in the future as the costs of cell expansion reduce.

#### 3.3.2 Tissue‐engineered medicines

There were 5 economic evaluations of ACI. One was a cost‐effectiveness analysis,^24^ which reported that a 1‐point increase in clinical scores (patient reported outcome measures) had lower costs for microfracture (MF) than for ACI at 5 years. Among the cost–utility analyses, Gerlier et al. (2010)^25^ showed CondroCelect to be cost‐effective compared with MF with an ICER of €16 229 per QALY gained. The main finding in Mistry et al. (2017)^27^ was that if the decision‐maker is willing to pay £20 000 for a QALY, ACI is 56–59% more likely to be cost‐effective than MF. Samuelson et al. (2012)^28^ estimated the *average* cost per QALY for periosteum‐covered ACI to be $9466 compared with $9243 for collagen‐covered ACI; no ICERs were presented. De Windt et al. (2018)^29^ compared single‐stage cartilage repair (instant allogeneic mesenchymal stromal [stem] cells product accompanying autologous chondron transplantation) with microfracture, and estimated the ICER to range from €28 588 to €147 513 per QALY gained. However, compared with ACI, the single‐stage procedure was forecast to be cost saving over a 5‐year horizon, largely as the cell expansion procedure is rendered redundant.

McAteer et al. (2007)^26^ utilised the headroom method to guide investment decisions in regenerative medicine. Based on tissue engineering applications in the urinary tract, they estimated a headroom of around £16 268, but noted the limited market, which may reduce potential profitability.

#### 3.3.3 Gene therapy medicines

The cost effectiveness of Strimvelis was examined in 2 analyses, of which 1 was deemed to be cost effective. South et al. (2018)^32^ reported a NICE Highly Specialised Technology Evaluation which estimated the most plausible ICERs for Strimvelis to be lower than £100 000 per QALY gained. NICE approved Strimvelis for the treatment of adenosine deaminase severe combined immunodeficiency, where a matched related donor is unavailable.^31^ In the treatment of severe haemophilia A, Machin et al. (2018)^30^ found that gene therapy is likely to be cost saving compared with the current standard of care involving FVIII prophylaxis. Zimmerman et al. (2019)^33^ estimated the ICER for voretigene neparvovec (Luxturna) for the treatment for vision loss owing to the ultra‐rare RPE65‐mediated inherited retinal disorders, at $480 100 per QALY gained. This was driven largely by the high cost of treatment and the relatively low gains in QALYs (1.3 over a lifetime), consistent with treatments that are neither *curative* nor extend life expectancy.

### 3.4 Quality of reporting

In terms of reporting, 13 studies^13^, ^14^, ^18^, ^19^, ^20^, ^21^, ^22^, ^25^, ^27^, ^29^, ^30^, ^33^ were deemed to be of good quality (see Supplementary Appendix S1). However, many were incomplete with respect to important methodological detail. The perspective was unclear in 7 of the studies.^15^, ^16^, ^24^, ^26^, ^28^, ^31^, ^32^ Two studies^15^, ^16^ did not state explicitly the modelling approach. Three studies^24^, ^27^, ^28^ did not mention explicitly a time horizon. Four studies^15^, ^16^, ^20^, ^29^ did not specify whether costs and outcomes were discounted. The reporting of sensitivity analysis was more complete, with evidence of deterministic univariate sensitivity analysis and multivariate probabilistic sensitivity analysis having been conducted in the majority of studies, with only 2^24^, ^26^ not mentioning any sensitivity analysis. While reporting quality was not analysed by study attributes, such as authorship affiliation, grey *vs* standard literature or country of origin, there were instances of high variability even within one reporting organisation. Variability in the quality of reporting of manufacturers' submissions to health technology agencies, as one example, is likely to be a function of what can be disclosed publicly, the level of detail provided by the manufacturer as well as the reporting template used. It is important to recognise that reporting quality may not reflect methodological quality.

### 3.5 Methodological challenges

#### 3.5.1 Size and design of trials

A recurring theme in the literature relates to the small size of clinical trials and the methodological challenges this presents. All ATMPs to date are indicated for rare diseases, which presents a challenge in terms of patient recruitment but, nonetheless, trials risk being statistically underpowered. Aae et al. (2018)^24^ highlighted the small sample sizes in trials, which might increase the risk of false negative findings, but perhaps equally important, also reduces the precision of the estimate of treatment effectiveness. Further evidence, including from post‐approval studies (e.g. Lam et al. 2019)^34^ are necessary to reduce uncertainty in key clinical parameters.

#### 3.5.2 Lack of data on disease progression and long‐term effects

Sarkar et al. (2019)^14^ discussed how CAR T‐cell therapy is a new therapy and so long‐term data on survival, costs, the role of HSCT after CAR T‐cell therapy and complications that could affect the cost effectiveness analysis results are lacking. Mistry et al. (2107)^27^ noted that the length of follow‐up in the published trials of chondral defect in the knee was too short and hence there are no long‐term data on success and failure rates. Further, because of the paucity of data from clinical studies, transition probabilities may not be calculable for parameterising economic models.

#### 3.5.3 Assumptions about efficacy and comparative effectiveness

Many economic evaluations required strong assumptions about the efficacy and comparative effectiveness of the ATMP, mainly due to the limitations of the available clinical evidence. In Machin et al. (2018),^30^ for instance, the assumption that successful gene therapy results in full quality of life was not substantiated by evidence, and could introduce significant bias in their estimates of cost‐effectiveness. Lin et al. (2018)^22^ stated, as a limitation, that no high‐quality long‐term clinical outcomes data existed for tisagenlecleucel. Some evaluations pertained to early phases of drug development, or were analyses of hypothetical drugs with very limited (if any) evidence on treatment effect. No randomised controlled trial data were available to Retel et al. (2017),^13^ for instance, and therefore data on the effectiveness of TIL had to be drawn from alternative, lower quality evidence.^35^, ^36^ A lack of comparative evidence limited the economic evaluation of Tice et al. (2018)^19^ and, as evidence on long‐term survival was largely unknown, further assumptions had to be made in relation extrapolating beyond the available evidence. The main limitation in Gerlier et al. (2010)^25^ was that a Markov model could not be constructed due to there being no robust data on the probability and time to occurrence of clinical events associated with osteoarthritis and total knee replacement. The absence of data was the main limitation also in Tappenden et al. (2010),^18^ where there was no randomised controlled trial evidence to input into the model; and Vilsboll et al. (2018)^20^ who reported a lack of uniform reporting tools to define the outcome of stress urinary incontinence interventions. Where strong evidence was not available, authors often relied on expert opinion. In the NICE (2016)^31^ review of whether their current methods of economic evaluation are *fit for purpose* in assessing ATMPs, they used hypothetical datasets to assess CAR T‐cell therapy in terms of a bridge to stem cell transplantation and with curative intent. They used theoretical prices that would result in the therapies being valued at the NICE willingness to pay thresholds of cost‐effectiveness. Overall, they found that while current NICE methods and processes were indeed robust and relevant for the appraisal of ATMPs, quantification of clinical outcomes and uncertainty were key to their evaluation.

#### 3.5.4 Lack of data on health‐related quality of life/utilities

The NICE (2017)^27^ assessment highlighted the limitation of relying on external data on patient quality of life. Similarly, Samuelson et al. (2012)^28^ noted a lack of available evidence and resorted to obtaining data on health state utility, as well as outcome scores, graft hypertrophy and failure rates from the literature. Mistry et al. (2017)^27^ also report a lack of evidence on utility values that could introduce additional uncertainty and potential bias. An absence of reliable data on utilities undermines the robustness of QALY calculations.

#### 3.5.5 Generalisability

The main themes in terms of generalisability relate to costs. Costs of ATMPs obtained from specific hospitals in specific countries, for instance, might limit generalisability to other jurisdictions.^12^, ^13^, ^24^, ^28^, ^29^ This may be due to different methods of production, pricing and service delivery in different settings. Other issues of generalisability highlighted in the reviewed studies, include the transferability of results from a US to a UK setting,^19^ the importance of age as a variable in potentially curative treatments^30^ and using QALYs based on the same multi‐attribute health status classification system internationally.^20^

### 3.6 Analysts' resolution of methodological challenges

The main methodological challenge was the lack of clinical data with which to inform any modelling or economic evaluation attempted.^12^, ^14^, ^18^, ^20^, ^22^, ^24^, ^25^, ^27^, ^28^ In all these studies, the problem was addressed by recourse to the published literature, or by making assumptions. For example, Mistry et al. (2017)^27^ derived transition probabilities from 2 studies, which compared matrix‐applied chondrocyte implantation with MF, and expert clinical opinion. Tice et al. (2018)^19^ estimated the time at which long‐term survivors would be considered effectively cured based on assumptions that were necessary to extrapolate the survival curve for trial participants. While disease modelling provides a way of estimating long‐term effects, this does not substitute for good quality clinical trial evidence.

## 4 DISCUSSION

### 4.1 Statement of principal findings

Of the 23 studies identified, 4^12^, ^13^, ^17^, ^30^ had interventions that dominated the comparator (more effective, and cost‐saving), while 2^18^, ^20^ estimated ICERs, which indicated that the interventions were dominated by the comparator treatment. The remaining studies had ICERs ranging from £14 395 per QALY gained (for autologous chondrocyte implantation) in Mistry et al. (2017),^27^ to USD$610 600 per QALY gained for instant allogeneic mesenchymal stromal cells product accompanying.^29^ The narrative overview of the methodological challenges encountered in the identified papers revealed as the principal difficulties, the paucity of trial data to inform economic analysis, the lack of long‐term data on outcomes and costs, and dependence on critical and often unsubstantiated assumptions. The clinical evidence was insufficient in many (if not most) instances to support claims that treatment was curative, which has a major bearing on estimates of survival and quality‐adjusted life expectancy required for calculating cost‐effectiveness.

### 4.2 Strengths and weaknesses of this review study

The main strength of this review is that it brings together an array of literature concerning the economic evaluation of ATMPs and identifies, from the studies, the main methodological challenges. The search terms were designed to have the maximum likelihood of identifying relevant articles; however, there are likely to be many unpublished economic evaluations submitted to HTA organisations, and presented at conferences (although abstracts were excluded explicitly), which were not included in the review. Our language restriction is a further limitation that excluded economic analyses published (or available from HTA organisations) in languages other than English.

### 4.3 Unique features of ATMPs for HTA

Although current methods of economic evaluation are considered by some organisations to be sufficient for analysing ATMPs,^5^, ^31^ there may be some unique features of ATMPs that require consideration when performing such analyses. Hettle et al. (2017),^5^ for instance, claim the factors that make ATMPs unique as the following: the potentially curative nature of the therapies along with lifetime benefits; the changing nature of the product characteristics over time; potential long‐term safety issues; organisational and scaling issues; and the significant up‐front cost that face payers.

Whether indeed these are unique to ATMPs is debatable (many surgical interventions have high up‐front costs with lasting benefits; antimicrobial treatments are curative; several medicines have potential long‐term safety concerns etc.). However, their exceptionally high costs demand higher evidential standards for claims of survival benefits and cure. The issue of whether or not certain ATMPs are curative is still not borne out in the literature. For tisagenlecleucel, the SMC (2019)^16^ assumed it to be curative if individuals in the study survived past 24 months. None of the economic evaluations included a value of information analysis to quantify the potential value of longer and larger trials to support the evidence base.

The differential timing in the costs and accrual of benefits associated with ATMPs suggests that time preference, and the choice of discount rate, is likely to be more impactful on their cost‐effectiveness compared to many other conventional health technologies. NICE (2017)^31^ applied a discount rate of 1.5% per annum for costs and benefits, in accordance with its guidance for treatments that restore people to full or near‐full health when they would otherwise die.^37^ Gerlier et al. (2010)^25^ highlighted a particular problem in their evaluation of the ATMP, ChondroCelect. Their application of a higher discount rate for costs than for effects meant that when the need for total knee replacement among patients with osteoarthritis receiving ChondroCelect increased, the ICER reduced in favour of ChondroCelect. However, the best treatment for the patient is the 1 that minimises pain and discomfort and avoids the need for knee replacement in the first place. This type of paradox could be encountered in other contexts and should be taken into consideration when conducting economic evaluations of ATMPs.

## 5 CONCLUSION

This systematic review is a comprehensive account and methodological critique of economic evaluations of ATMPs. In particular, it provides a narrative synthesis of the challenges facing health technology analysts and economists in the evaluation of ATMPs. The main issue identified was the paucity of long‐term clinical trial data to inform cost effectiveness analyses. This was the case in 11 of the 23 papers identified. Analysts had to resort to strong assumptions about the curative nature of ATMPs and their ability to return patients to full health‐related quality of life. Such assumptions can lead to biased estimates of cost‐effectiveness and inefficient allocation of resources. There are also implications for the funding of ATMPs, especially in terms of outcomes‐based payment, which depends critically on the measurement of treatment success.

## COMPETING INTERESTS

H.Ll.‐W. and D.A.H. declare that they have no conflict of interest.

## CONTRIBUTORS

H.Ll.‐W. and D.A.H. contributed substantially to the conception and design of the work. All authors made contributions to the acquisition, analysis, or interpretation of data. H.Ll.‐W. drafted the paper and all authors revised it critically for important intellectual content, and gave their final approval of the version to be published. D.A.H. agrees to be accountable for all aspects of the work in ensuring that questions related to the accuracy or integrity of any part of the work are appropriately investigated and resolved.

## Supporting information


**APPENDIX S1** Quality reporting using Consolidated Health Economic Evaluation Reporting Standards [10]
