# Supplementary material for: A systematic review of economic evaluations of advanced therapy medicinal products
Source: Br J Clin Pharmacol. 2020 Mar 31;87(6):2428–43. doi: 10.1111/bcp.14275 (PMC8247439; doi:10.1111/bcp.14275)
Supplement: Supplementary file 1 — APPENDIX S1 Quality reporting using Consolidated Health Economic Evaluation Reporting Standards [10] [file BCP-87-2428-s001.docx]

**Supplementary Appendix:** Quality Reporting using CHEERS [10]

| Reference → | [28] | [13] | [5] | [14] | [15] | [29] | [16] | [17] |
| --- | --- | --- | --- | --- | --- | --- | --- | --- |
| Title | 0 | 1 | 1 | 0 | 1 | 1 | 1 | 0 |
| Abstract | 1 | 1 | 1 | 0 | 0 | 1 | 1 | 0 |
| Background and objectives | 0 | 1 | 1 | 1 | 1 | 0 | 1 | 1 |
| Target population and subgroups | 0 | 0 | 1 | 1 | 1 | 0 | 1 | 1 |
| Setting and location | 0 | 0 | 1 | 0 | 0 | 0 | 0 | 1 |
| Study perspective | 0 | 1 | 1 | 1 | 1 | 0 | 1 | 0 |
| Comparators | 1 | 1 | 1 | 1 | 1 | 1 | 1 | 0 |
| Time horizon | 1 | 1 | 1 | 1 | 1 | 0 | 1 | 0 |
| Discount rate | 1 | 1 | 1 | 1 | 1 | 0 | 1 | 1 |
| Choice of health outcomes | 1 | 1 | 1 | 0 | 1 | 0 | 1 | 1 |
| Measurement of effectiveness | 1 | 1 | 1 | 0 | 1 | 0 | 1 | 1 |
| Measurement and valuation of preference-based outcomes | 1 | 1 | 1 | 1 | 0 | 0 | 0 | 0 |
| Estimating resources and cost | 1 | 1 | 1 | 1 | 1 | 1 | 1 | 1 |
| Currency, price date and conversion | 1 | 1 | 1 | 0 | 0 | 0 | 1 | 1 |
| Choice of model | 1 | 1 | 1 | 1 | 1 | 1 | 1 | 1 |
| Assumptions | 1 | 1 | 1 | 1 | 1 | 1 | 1 | 1 |
| Analytic methods | 1 | 1 | 1 | 1 | 1 | 1 | 1 | 0 |
| Study parameters | 1 | 1 | 1 | 0 | 1 | 1 | 1 | 0 |
| Incremental costs and outcomes | 0 | 1 | 1 | 1 | 1 | 0 | 1 | 1 |
| Characterising uncertainty | 0 | 1 | 1 | 1 | 1 | 0 | 1 | 1 |
| Characterising heterogeneity | 0 | 0 | 0 | 0 | 0 | 0 | 1 | 0 |
| Study findings, limitations, generalizability and current knowledge | 1 | 1 | 1 | 1 | 1 | 0 | 1 | 0 |
| Source of funding | 1 | 1 | 0 | 0 | 1 | 1 | 1 | 1 |
| Conflict of interest | 1 | 1 | 1 | 0 | 1 | 1 | 1 | 1 |
|  |  |  |  |  |  |  |  |  |
| Mean score (%) | 66.7 | 87.5 | 91.7 | 58.3 | 79.2 | 41.7 | 91.7 | 58.3 |

| Reference → | [18] | [19] | [20] | [21] | [22] | [23] | [24] |  |  |  |  |
| --- | --- | --- | --- | --- | --- | --- | --- | --- | --- | --- | --- |
| Title | 1 | 1 | 1 | 0 | 0 | 0 | 0 |  |  |  |  |
| Abstract | 1 | 1 | 1 | 0 | 0 | 0 | 1 |  |  |  |  |
| Background and objectives | 1 | 1 | 1 | 1 | 1 | 1 | 1 |  |  |  |  |
| Target population and subgroups | 0 | 0 | 0 | 0 | 0 | 0 | 0 |  |  |  |  |
| Setting and location | 0 | 0 | 0 | 0 | 0 | 0 | 0 |  |  |  |  |
| Study perspective | 1 | 0 | 1 | 0 | 0 | 0 | 1 |  |  |  |  |
| Comparators | 1 | 1 | 1 | 1 | 1 | 1 | 1 |  |  |  |  |
| Time horizon | 1 | 1 | 0 | 1 | 1 | 1 | 1 |  |  |  |  |
| Discount rate | 1 | 1 | 1 | 0 | 0 | 1 | 1 |  |  |  |  |
| Choice of health outcomes | 1 | 1 | 1 | 1 | 1 | 1 | 0 |  |  |  |  |
| Measurement of effectiveness | 1 | 1 | 1 | 1 | 1 | 0 | 0 |  |  |  |  |
| Measurement and valuation of preference-based outcomes | 1 | 0 | 1 | 1 | 1 | 1 | 1 |  |  |  |  |
| Estimating resources and cost | 1 | 1 | 1 | 1 | 1 | 1 | 1 |  |  |  |  |
| Currency, price date and conversion | 1 | 0 | 0 | 0 | 0 | 0 | 1 |  |  |  |  |
| Choice of model | 1 | 1 | 1 | 0 | 0 | 1 | 1 |  |  |  |  |
| Assumptions | 1 | 1 | 1 | 0 | 0 | 1 | 1 |  |  |  |  |
| Analytic methods | 1 | 1 | 1 | 0 | 0 | 1 | 1 |  |  |  |  |
| Study parameters | 1 | 0 | 1 | 0 | 0 | 0 | 1 |  |  |  |  |
| Incremental costs and outcomes | 1 | 1 | 1 | 1 | 1 | 1 | 1 |  |  |  |  |
| Characterising uncertainty | 1 | 1 | 1 | 1 | 1 | 1 | 1 |  |  |  |  |
| Characterising heterogeneity | 0 | 0 | 0 | 0 | 0 | 0 | 0 |  |  |  |  |
| Study findings, limitations, generalizability and current knowledge | 1 | 1 | 1 | 0 | 0 | 1 | 1 |  |  |  |  |
| Source of funding | 1 | 1 | 1 | 0 | 0 | 1 | 0 |  |  |  |  |
| Conflict of interest | 1 | 1 | 1 | 0 | 0 | 1 | 0 |  |  |  |  |
|  |  |  |  |  |  |  |  |  |  |  |  |
| Mean score (%) | 87.5 | 70.8 | 79.2 | 37.5 | 37.5 | 62.5 | 66.7 |  |  |  |  |

| Reference → | [25] | [26] | [30] | [31] | [32] | [27] | [36] | [37] |
| --- | --- | --- | --- | --- | --- | --- | --- | --- |
| Title | 1 | 1 | 1 | 1 | 0 | 1 | 1 | 1 |
| Abstract | 0 | 0 | 1 | 0 | 1 | 1 | 1 | 1 |
| Background and objectives | 1 | 1 | 1 | 1 | 1 | 1 | 1 | 0 |
| Target population and subgroups | 1 | 1 | 0 | 0 | 0 | 1 | 1 | 0 |
| Setting and location | 0 | 0 | 1 | 0 | 0 | 1 | 1 | 1 |
| Study perspective | 1 | 1 | 1 | 1 | 1 | 1 | 1 | 1 |
| Comparators | 1 | 1 | 1 | 1 | 1 | 1 | 1 | 1 |
| Time horizon | 1 | 1 | 1 | 1 | 1 | 1 | 1 | 1 |
| Discount rate | 1 | 1 | 0 | 1 | 1 | 1 | 1 | 0 |
| Choice of health outcomes | 1 | 1 | 1 | 1 | 1 | 1 | 1 | 1 |
| Measurement of effectiveness | 1 | 1 | 1 | 1 | 1 | 0 | 0 | 1 |
| Measurement and valuation of preference-based outcomes | 1 | 1 | 1 | 1 | 1 | 1 | 1 | 0 |
| Estimating resources and cost | 1 | 1 | 1 | 1 | 1 | 1 | 1 | 1 |
| Currency, price date and conversion | 0 | 1 | 0 | 0 | 0 | 1 | 1 | 0 |
| Choice of model | 1 | 1 | 1 | 1 | 1 | 1 | 1 | 1 |
| Assumptions | 1 | 1 | 1 | 1 | 1 | 1 | 1 | 0 |
| Analytic methods | 1 | 1 | 1 | 1 | 1 | 1 | 1 | 1 |
| Study parameters | 1 | 1 | 1 | 1 | 1 | 0 | 0 | 1 |
| Incremental costs and outcomes | 1 | 1 | 1 | 1 | 1 | 1 | 1 | 1 |
| Characterising uncertainty | 1 | 1 | 1 | 1 | 1 | 1 | 1 | 1 |
| Characterising heterogeneity | 0 | 0 | 0 | 0 | 0 | 0 | 0 | 0 |
| Study findings, limitations, generalizability and current knowledge | 1 | 1 | 1 | 1 | 1 | 1 | 1 | 1 |
| Source of funding | 0 | 0 | 0 | 0 | 1 | 1 | 0 | 0 |
| Conflict of interest | 1 | 1 | 1 | 1 | 1 | 0 | 0 | 0 |
|  |  |  |  |  |  |  |  |  |
| Mean score (%) | 79.2 | 83.3 | 79.2 | 75.0 | 79.2 | 83.3 | 79.2 | 62.5 |
